# Exploring perspectives of older adults and informal caregivers on physical activity during non-weight-bearing rehabilitation: a qualitative study

**DOI:** 10.1007/s41999-025-01390-x

**Published:** 2026-01-08

**Authors:** Elma van Garderen, Mandy Visser, Wilco P. Achterberg

**Affiliations:** 1 Department of Public Health and Primary Care, Leiden University Medical Center, Hippocratespad 21, 2333 ZD Leiden, The Netherlands; 2 Topaz, Aaltje Noordewierlaan 50, 2324 KS Leiden, The Netherlands

**Keywords:** Non-weight-bearing, Older adults, Inpatient rehabilitation, Informal caregiver, Physical activity

## Abstract

**Background:**

Older adults in inpatient rehabilitation often struggle to remain physically active, especially when restricted from bearing weight on a lower extremity. Given the negative consequences of inactivity, it is important to provide and encourage physical activities and include patients’ and informal caregivers’ input and views. This qualitative study explores the perspectives of patients receiving non-weight-bearing rehabilitation and their informal caregivers regarding the content and frequency of physical activities, and the motivational factors for performing such physical activities.

**Methods:**

Semi-structured interviews were conducted with ten patients (8 female, aged 61–87) restricted from weight-bearing on a lower extremity, primarily due to fractures, and six informal caregivers (3 female, aged 58–86). The interviews were thematically analyzed using the framework method.

**Results:**

Eleven themes emerged regarding physical activity during the non-weight-bearing period. The themes describe 1) the interpretation of physical activity during the non-weight-bearing period; 2) activity limitations; 3) frequency, duration, and 4) content of physical therapy; 5) patient-regulated exercise; 6) preferences for group versus individual therapy; 7) the healthcare providers, and 8) informal caregivers’ role in facilitating and encouraging physical activity; 9) informing the patient and informal caregiver; 10 the rehabilitation location; and 11) intrinsic motivation.

**Conclusion:**

Physical activity during the non-weight-bearing period should be tailored to the patient and their informal caregivers. It is important to inform and discuss the possibilities for being physically active with the patient. The most important goals for engaging in physical activity were regaining the ability to walk and returning home.

## Key summary points

**Aim:**

We conducted semi-structured interviews with older adults during non-weight-bearing rehabilitation and their informal caregivers regarding the content and frequency of physical activities, and motivational factors for performing such physical activities.

**Findings:**

Eleven themes emerged from the interviews, which corresponded to the domains of the ICF model.

Physical activity during the non-weight-bearing period should be tailored to the patients receiving rehabilitation and their informal caregivers.

**Message:**

We recommend integrating the perspectives of patients receiving non-weight-bearing rehabilitation and their informal caregivers into their rehabilitation plans and addressing any differences between their perspectives and those of healthcare providers.

## Introduction

It is challenging for older adults to remain physically active during inpatient care [1–3]. They spend most of the day sitting or lying down (23.1 h a day), and only 16% of daytime on physical activities, including self-care, therapy, and transferring [3, 4]. Inactivity has serious consequences. Five to 10 days of bed rest can significantly reduce muscle strength, muscle mass, physical condition, and motivation to resume physical activity [5, 6]. Prolonged periods of sedentary behavior and weight-bearing restrictions have even been linked to increased mortality [7, 8].

Restrictions in weight-bearing often stem from the need to protect healing tissues and ensure optimal recovery following lower extremity injuries or surgery. Physicians may advise a non-weight-bearing (NWB) period to prevent complications such as fracture displacement, delayed bone healing, or impaired wound healing. While this period may be important for recovery, being restricted from bearing weight on a lower extremity increases the challenge to remain physically active [9]. Yet, 98% of healthcare professionals agreed that it is important for patients restricted to NWB to remain physically active [10].

After hospital discharge, older adults with weight-bearing restrictions are often transferred to a temporary care facility, where they receive rehabilitation aimed at maintaining or restoring their physical functioning. Including the perspectives of both patients restricted to NWB and informal caregivers is essential for promoting physical activity during rehabilitation [11, 12]. Shared decision-making can enhance communication between healthcare providers and patients, improve the accuracy of patients’ expectations, and increase satisfaction with care [13]. Moreover, active involvement in setting and planning rehabilitation goals improves functional outcomes and goal attainment among patients [14]. The involvement of informal caregivers has been associated with better physical functioning, a higher level of independence and an increased likelihood of patients returning home [15–18]. Furthermore, their involvement reduces anxiety and depression and enhances quality of life for both patients and informal caregivers [15–18]. To prevent physical inactivity, it is important to understand which activities both patients restricted to NWB and their informal caregivers perceive as physical activities, which activities they find important, and what factors motivate or discourage them from remaining physically active.

However, evidence on how to support patients restricted to NWB in maintaining physically active is limited, and input from the patients and informal caregivers regarding their views of physical activity during the NWB period is lacking [7, 19]. This qualitative study therefore explores their perspectives on the content and frequency of physical activities, as well as the motivational factors that facilitate or hinder engagement. Such insights may inform the development and implementation of interventions aimed at promoting physical activity among patients restricted to NWB and preventing inactivity.

## Method

### Design

This qualitative study used an inductive descriptive approach to identify and describe topics of importance to the participants [20]. We adhered to the standards for reporting qualitative research [21]. The Medical Ethics Committee of the Leiden University Medical Center has declared that this study is not subject to the Medical Research Involving Human Subjects Act (WMO) (Protocol number 23–3125).

### Participant population and recruitment

This study used a convenience sampling method. We reached out to geriatric inpatient rehabilitation centers (GR centers) through the University Network of the Care sector South Holland (UNC-ZH network) and LinkedIn. Participating centers informed potential candidates, extended invitations, and facilitated contact between the candidates and the researcher (EvG). Informal caregivers were approached either directly by the patients restricted to NWB or, with their consent, by the researcher. Within the GR centers, patients restricted to NWB received individualized care tailored to their needs, which could include medical care, physiotherapy, occupational therapy, and other supportive services.

Eligible for inclusion were patients aged 60 + , who were temporarily admitted to a GR center and temporarily restricted from bearing weight on at least one lower extremity. Exclusion criteria for potential candidates were: an inadequate comprehension or production of the Dutch language, delirium, end-of-life care pathway, communication disability, psychiatric disorder. or cognitive impairment that would hinder conducting an interview. Informal caregivers were eligible for inclusion if their relative, the NWB patient, met the aforementioned inclusion criteria. They also needed to have an adequate comprehension and production of the Dutch language and no communication disability, or psychiatric disorder or cognitive impairment that would impede participation in an interview.

Potential participants received an information letter and were asked to sign the informed consent after ample time for consideration. We aimed to interview ten0 patients restricted to NWB and ten informal caregivers, based on the research group’s expectation that this sample size would provide sufficient data to address the research question [22].

### Data collection

Semi-structured interviews were conducted and recorded by EvG between April and October 2024. The interview guide covered five topics: general, knowledge, behavior, motivation, and content (Appendix 1). The guide was refined through discussions with a peer group consisting of representatives of patients, informal caregivers, and healthcare providers, and validated through pilot interviews with one NWB patient and one informal caregiver. The interviewer asked open-ended questions about each topic and in-depth follow-up questions to gain a better understanding. Verbal summaries were provided throughout the interview giving interviewees the opportunity to offer additional comments or corrections.

One-on-one interviews with patients restricted to NWB were conducted at GR centers. One-on-one interviews with informal caregivers were held at GR centers, by telephone, or at a location convenient for the informal caregiver. The interviews took place in the pre-determined predicted last week of the NWB period to ensure participants had relevant experience while still in that situation. The interviews lasted approximately 45 min.

### Data analysis

The interviews were thematically analyzed using the framework method [23]. The data analysis was performed by EvG and MV. EvG is a female physiotherapist and PhD student with formal training in qualitative research and 7 years of experience in geriatric rehabilitation. MV is a female experienced qualitative researcher. We followed the seven stages of the framework method [23]: 1) The interviews were transcribed verbatim in Dutch. 2) EvG and MV familiarized themselves with the interview data. 3) Both researchers openly coded the first two transcripts of both the patients restricted to NWB and their informal caregivers; EvG continued coding all remaining transcripts. 4) EvG and MV compared their codes and grouped them into themes to construct the initial framework. 5) EvG used the framework to analyze the remaining transcripts. 6) Any new codes or themes that emerged were added to the framework. 7) Interpreting the data. The Atlas.ti program was used for coding and developing the analytical framework. After the final stage, interpreting the data, the results were translated to English and reported. Relevant quotes were translated into English by a native speaker often consulted by the Leiden University Medical Center. To provide a clear description of the participants, the characteristics of the patients restricted to NWB and informal caregivers, such as age, gender, duration, and reason for the NWB period, were gathered.

## Results

### Characteristics

Ten patients restricted to NWB and seven informal caregivers from four GR centers consented to be interviewed. One informal caregiver interview was excluded due to a recording failure. Although all patients agreed to participate, most declined permission to contact their informal caregiver, citing time constraints or limited involvement. Consequently, only two caregiver interviews were directly linked to participating patients. Recruitment of informal caregivers continued after the inclusion of the ten patients, but concluded after six interviews due to data sufficiency and time constraints. The patients were between 61 and 87 years old and the informal caregivers between 58 and 86 years old. Most patients were restricted in bearing weight on a lower extremity due to one or more fractures of the lower extremity. Characteristics of the participants are shown in Table 1.

**Table 1:** Demographic characteristics of the participating non-weight-bearing patients and informal caregivers

	Patients *n* = 10 (100%)	Informal caregivers *n* = 6 (100%)
Gender, n (%)		
Female	8 (80%)	3 (50%)
Age range (median)	61–87 (71)	58–86 (76)
Weeks since advised NWB median (IQR)	7 (5.00–9.75)	5.5 (5.00–7.25)
Reason for admission to GR, N (%)	Unplanned surgery: 7 (70%)	* Unplanned surgery: 6 (100%)
	One ore more fractures in a lower extremity, 7 (70%)	One ore more fractures in a lower extremity, 6 (100%)
	Planned surgery: 3 (30%)	
	Amputation, 1 (10%)	
	Infection in hip prosthesis, 1 (10%)	
	Ankle osteoarthritis, 1 (10%)	

N: Number. NWB: Non-weigh- bearing. IQR: Interquartile range. GR: Geriatric rehabilitation. *Reason for admission to GR of the NWB patient for whom they are the informal caregiver

The level of pre-hospitalization physical activity varied from participating in sports activities to being inactive due to a history of falls or pain. However, both the pre-hospitalization active and inactive patients experienced loss of strength, condition, and independence during their hospitalization. All patients received medical and therapeutic care during their stay in the GR centers.

### ICF model

**Fig. 1:**
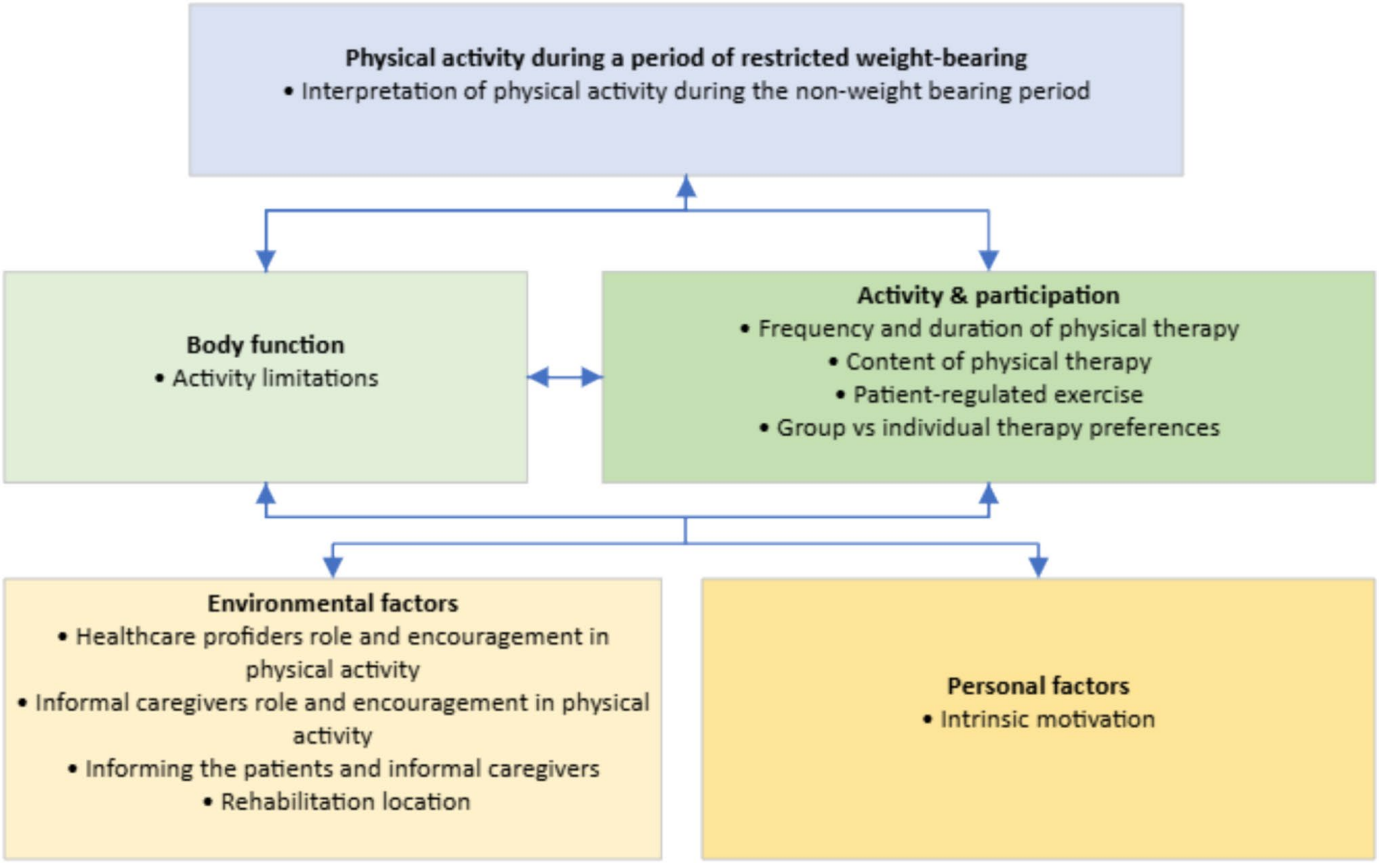
ICF model physical activity during a period of restricted weight-bearing of a lower extremity

The ICF model provides a comprehensive framework for understanding health and health-related domains [24]. While analyzing the interviews, it became evident that the identified themes corresponded with the domains of the ICF model (Fig. 1). Figure 1 should not be interpreted as a conceptual model; rather, it illustrates how the themes were organized according to the ICF framework.

*Body function:* Body functions or structures motivating or obstructing physical activity.

*Activity and participation:* (Preferred) execution of physical activity by an individual and participation in social life.

*Environmental factors:* External factors influencing physical activity.

*Personal factors:* Internal factors influencing physical activity.

In the context of this study, the ICF model's domain of health refers to physical activity during a period of restricted weight-bearing on a lower extremity for older adults. Given the importance of understanding what qualifies as physical activity during this NWB period, we examine participants' interpretation of physical activity—which activities do they personally define as physical activities.

## Physical activity during a period of restricted weight-bearing

### Interpretation of physical activity during the non-weight-bearing period

The participants had different views on what qualifies as being physically active during the NWB period. Some considered physical activity impossible during this time.

*I feel completely blocked, normal movement is at least walking, basically being able to do everything, like going to the bathroom on my own, getting up by myself, and so on. (Patient 3, female, aged 87).*

Most participants, however, considered strength training and other exercises given by the physiotherapist to be physical activities, followed by performing activities of daily living (ADL) and self-propelling the wheelchair.

*Exercise. That means stretching your leg. So making sure your leg muscles stay strong, your back, things like that. I move my arms a lot because I use a self-propelled wheelchair. And then sometimes I also use my foot, sometimes I don't. Because then I throw them both in the air, and then I think: right, and a leg exercise as well. (Patient 10, female, aged 66).*

According to one participant, self-propelling the wheelchair only counts as physical activity if it is challenging for the arms. Most participants experienced limited possibilities in performing physical activities during the NWB period .

## Body function

### Activity limitations

The participants experienced limited ability to perform physical activities due to the weight-bearing restrictions, (pre-)existing comorbidities, multiple fractures, and low energy due to poor sleep. Pain, fear of falling, and concern about the wound not healing properly were reasons participants gave for being cautious when they performed physical activities.

*So I say, we're not going to work on that, because it will go, it will get worse again. And it's my body, not yours, so when I say, I'm not doing that, then I'm not doing it. Plain and simple. (Patient 7, male, aged 70).*

## Activity and participation

### Frequency and duration of physical therapy

Most of the participating patients mentioned receiving some form of physical therapy between two and four times a week for 30 min per session. Most patients found this sufficient, while some preferred more frequent therapy or greater access to the fitness room and more patient-regulated exercises. Informal caregivers indicated that 30 min was sufficient, but suggested adjusting the time based on the patient's fitness level. However, they considered the number of sessions inadequate.

*Yes, yes, yes, and we were also, well not surprised, but gosh, she only has physiotherapy two or three times a week, is that enough, you know, so you think, well, can' they do half an hour of exercise every day. (Informal caregiver 1, female, aged 58).*

### Content of physical therapy

Physical therapy during the NWB period should, according to most participants, include some form of strength training. They noted that physical therapy already includes, or should include, standing and ‘hopping’ exercises using a walking frame or parallel bars, wheelchair mobilization, seated whole-body exercises, and arm or single-leg cycling. Particularly popular among participants was cycling, combined with a virtual reality video screen. Some of the participants reported that making the physical exercises enjoyable stimulates their participation. Activities or components they mentioned which made physical exercises enjoyable included: cycling (using arms or one leg) with a virtual reality video screen, incorporating variation, adding playful or competitive elements, and performing exercises while watching TV or outdoors.

And with a video clip to watch. Then you're on holiday for 15 min, you know? It's true, isn’t it? (Patient 8, female, aged 80).

### Patient-regulated exercise

Data shows that patient-regulated exercises include wheelchair mobilization, standing on one leg, strength training for the legs or arms, stretching and mobilizing exercises for various body parts, and performing activities of daily living (ADL) such as watering plants.

*And sometimes then I also look for it like, well, there aren't that many flowers now, but I used to have a house full of flowers, and then I'd have to water them and clean up, and I'd be back and forth five times with a small cup—haha like that. (Patient 5, female, aged 66).*

Some patients mentioned having their own repertoire of exercises, while others stated that they received or requested exercises from physio- or occupational therapists. Most patients reported performing their exercises daily. The duration and frequency varied among patients, ranging from continuous activity throughout the day to short sessions of 5 min several times per day, or a single 15-min session daily.

Yes, I try to do small exercises with clockwork regularity to stay as active as possible. (Patient 3, female, aged 87).

Only two patients mentioned that whether they performed the patient-regulated exercises on a specific day depended on other factors such as how they felt, pain levels, and other activities they had planned for that day, including physical therapy.

### Group vs. individual therapy preferences

Group therapy was generally favored by patients, sometimes combined with individual therapy. The informal caregivers, however, believed that the patients mostly preferred individual therapy.

*She highly appreciates the physiotherapy she receives. She values it greatly, she asks specific questions and the physiotherapist focuses on her abilities and how well she’s progressing. So individualized physiotherapy." (Informal caregiver 2, male, aged 66).*

Participants expressed that individual therapy should focus on solutions, i.e., performing ADL independently, and that the focus of group therapy should be on general training. According to participants, the merits of group therapy are: the therapists do not hover around you, you can chat with others, it makes the therapy more enjoyable, it distracts you, and it makes you humble.

*Well, sometimes a group lesson is also fun because you are with others who are in the same boat, and you also challenge each other a bit or look at what others are doing. Like, oh can you do that as well, I didn't know that, can I do that too? You know, like you're kind of learning by copying. (Patient 5, female, aged 66).*

## Environmental factors

### Healthcare providers’ role in facilitating and encouraging physical activity

According to the participants, physiotherapists play an important role in facilitating physical activity. Their role includes: providing therapy sessions; instructing patients on how to perform exercises, and on frequency and duration of patient-regulated exercises; installing the exercise equipment; educating patients about physical (in)activity and its consequences; providing solutions for performing ADL independently; ensuring safety and taking responsibility for the exercises performed by the patients.

*But the other thing is, when it comes to movement, I still think you have to limit that to the, to where the person, or, to what the physiotherapists can monitor. (Patient 9, male, aged 72).*

Occupational therapists were described as being responsible for providing or repairing wheelchairs and assisting with the transition home by offering guidance on assistive devices and training in ADL, such as cooking. Participants noted that this assistance usually begins later in the rehabilitation process, typically when patients are permitted to bear weight on both legs.

The role of nurses was primarily associated with assistance in ADL, with their involvement varying according to the patient’s level of independence and energy. Participants indicated that more help is often required at the start of the rehabilitation and for patients with low energy levels. As the patients become more independent, the nurses’ involvement can decrease.

*I hardly see the nurses, actually. In the evening they come to bring the bedpan and then it's "oh, you're in bed already, well bye! Haha see you tomorrow! (Patient 4, female, aged 62).*

Participants described that the level of encouragement required from various healthcare providers depends on the individual patient. Some patients expressed that a stricter approach could be beneficial in achieving their goals. Reported methods to encouraging physical activity include providing group therapy, promoting independence in ADL and patient-regulated exercises, monitoring the execution of these exercises, educating patients, and emphasizing the consequences of physical inactivity.

I, I think it's different for each person. Some people need a kick up the backside and others you need to leave alone. (Patient 10, female, aged 66).

### Informal caregivers' role in facilitating and encouraging physical activity

Some patients reported that their informal caregivers motivated them by complimenting their performance and offering encouragement. However, all informal caregivers reported that they did not encourage the NWB patient to be physically active. Reasons given for not encouraging physical activity include the patients’ own motivation or unwillingness to listen, and the informal caregivers’ perception that it is not their responsibility or that they are not sufficient involved. Moreover, all participants stated that informal caregivers were not involved in physical activities during the NWB period. Informal caregivers mentioned not being involved in physical activities because the NWB patient did not want or need help, others took over the care, or they themselves were not around enough to assist. Some informal caregivers chose not to be involved due to their own health issues, feelings of incompetence, believing that it is not their job, and because they already have to assist when the patient returns home.

When she’s at home, I have to help her. But not during the few hours that I’m here [ in the facility]. (Informal caregiver 3, male, aged 75).

Patients mentioned being reluctant in asking their informal caregivers for help. The reasons given were: they did not want to burden their informal caregiver too much, they believed that assisting with physical activities is not their responsibility, and they viewed their role as providing companionship and taking them outside and to places like restaurants.

*I don’t think it’s my family’s job to get me to do my exercises. More to take me along and do other things with me, but not like “Go sit on your chair and do it”. (Patient 3, female, aged 87).*

Informal caregivers emphasized the importance of being informed by the healthcare provider when their involvement is required. They stated that without this communication, the patient might not accept help or could feel uncomfortable asking their informal caregivers for assistance.

*Well, actually, actually from, from someone with more authority. Because she would say, 'no, there's no need, I can do that.' But if it comes from…, from a bit higher up, I think she would accept it more easily. (Informal caregiver 4, female, aged 86).*

### Informing the patients and informal caregivers

Some patients emphasized the importance of being adequately informed about the challenges of maintaining independence while adhering to weight-bearing restrictions.

*Well, what I didn’t fully realize, and no one really told me this either, you just find out in practice, is that you become completely dependent on others. Completely. (Patient 9, male, aged 72).*

They reported that they received information about not being allowed to bear weight on the affected leg, along with general or specific instructions regarding physical activities during the NWB period. Patients indicate that instructions for patient-regulated exercises should be provided verbally or in written form and should include pictures. Some participants preferred both options. Only one patient received information through e-health. Although she found it convenient, she did not prefer it over written instructions.

*You can watch it again and again, although the picture that was hanging there was also very handy. That showed everything at a glance, now I have to start it up. Because everything else I do on my own laptop. (Patient 10, Female, aged 66).*

Most informal caregivers mentioned that they did not receive information about physical activity. They did not feel the need to be informed because they trusted the professionals' capabilities, received information from the patient, or simply asked for information when needed.

*Well, she tells us. We just ask, “So, what are you allowed to do?” Sure, I could go with her to the physiotherapy room, but I don’t really feel like it. (Informal caregiver 4, female, aged 86).*

The informal caregivers expressed a preference for receiving information about the rehabilitation plan when the patient is allowed to bear weight again. They also wanted details about the process of the patient returning home, including which assistive devices to purchase.

### Rehabilitation location

Participants emphasized the importance of wheelchair accessibility at the rehabilitation location and its immediate surroundings. This includes having no thresholds or sloping floors, wheelchair-accessible elevators and (exit) doors, easy exit access, as well as smooth, even sidewalks outside.

*And what I think is really important: remove the thresholds in the buildings. Because if they don't place the wheelchair ramp for me, I can't get to the terrace here. (Patient 10, female, aged 66).*

## Personal factors

### Intrinsic motivation

Coping strategies reported for staying motivated despite experiencing fear, pain, exhaustion, or reluctance to perform physical activity include thinking ahead to when weight-bearing will be allowed again, doing what one is capable of, and maintaining a positive outlook (e.g., things could be worse). Participants expressed their hope that therapy can help manage fear.

*Yes, basically giving her back her confidence. That, to me, is the most important thing you can have. The people who treat her, restore that confidence. (Informal caregiver 3, male, aged 75).*

Several patients mentioned experiencing dependency at some point or throughout the entire NWB period. However, most participants emphasized the importance of being independent and trying to do as much as possible on their own.

*Yes of course. You have to arrange everything yourself, you know. You shouldn't press that button every time like, come and help me, you shouldn't do that of course. You have to try it yourself first, and if you really can't do it, well, then you should call someone, but you have to try everything yourself. That's motivation if you want to, like, progress. (Patient 7, male, aged 70).*

Patients reported that the social aspect, distraction, and a sense of accomplishment motivated them to remain physically active. Participants emphasized the importance of staying active to maintain or improve physical function during the NWB period, including strength, being able to sit up for longer periods of time, and physical condition. Other incentives mentioned included avoiding prolonged sitting, changing body positions to reduce stiffness and pain, improving balance, and achieving weight loss. The goals of performing physical activity viewed as most important were: regaining the ability to walk, returning home, and resuming life as they knew it, including family activities, sports, and traveling without limitations (e.g., driving a car).

I only have one motive, as you know. And that is that I want to walk out the door using both my legs. (Patient 8, female, aged 80).

## Discussion

In this article, we describe the perspectives of patients restricted to NWB and their informal caregivers regarding the content and frequency of physical activities, as well as the motivational factors affecting these activities. Eleven themes emerged from the interviews, which corresponded with the domains of the ICF model. The domain of health reflects the participants’ interpretation of physical activities during the NWB period, and the domain of body function captures activity limitations. Themes related to the domains of activity and participation include: frequency, duration, and content of physical therapy; patient-regulated exercise; and preferences for group versus individual therapy. Themes within the domains of environmental and personal factors include the healthcare providers’ and informal caregivers’ role in facilitating and encouraging physical activity, the dissemination of information, the rehabilitation location, and intrinsic motivation.

### Frequency of physical activity

Despite previous evidence of inactivity among older adults during inpatient care, our findings suggest that meeting physical activity guidelines may still be achievable [1–4]. Most patients received physical therapy two to four times a week for 30 min, combined with patient-regulated exercises. If these activities were of moderate or vigorous intensity, they may have met the physical activity guidelines [25]. Nevertheless, it is important to avoid long periods of sedentary behavior between exercise sessions.

### Motivational factors

Understanding the motivational factors can help encourage physical activity. Behavior is influenced by knowledge, awareness, and attitude. Barriers of physical activity include unwillingness to move, physical health status, symptoms, and fear [26]. These barriers are consistent with those identified during the NWB period. Motivating physical activity involves removing or addressing these barriers. This study highlights the important role of healthcare providers, particularly physiotherapists, in promoting and facilitating physical activity. Methods they can use, identified in this study, include educating patients, encouraging independence in ADL, providing group therapy, making therapy enjoyable (e.g., using e-health), and offering patient-regulated exercises. Healthcare providers' responsibility to promote physical activity may decrease as patients become more independent [27]. Another important motivator is found in the patients’ own coping strategies and short- and long-term goals. Involving participants in goal setting is found important in GR, because it leads to patient-centered care, even if it does not necessarily improve physical function [28, 29]. While supporting and involving informal caregivers are seen as important promotors of physical activity [26], our study found that their involvement was almost nonexistent. This indicates a gap between the expectations of the caregivers’ role and reality, as well as potential selection bias [30, 31]. Participants mentioned a lack of information—which they generally did not mind—and a lack of involvement, due to the rehabilitation center assuming responsibility of care. This reduced involvement may also reflect the high caregiver burden experienced by informal caregivers, which can limit their capacity to engage in additional activities such as promoting physical activity [32–34].

## Strengths and limitations

To the best of our knowledge, this is the first study to explore the perspectives of patients and their informal caregivers regarding physical activity during the NWB period. A strength of this study is the timing of the interviews, which were conducted during the final week of the predetermined NWB period. This ensured that the participants had experience with the NWB period while still being in that situation. Another strength is that we interviewed patients from four rehabilitation centers, making sure that the outcomes are not location-specific, increasing transferability.

We had difficulty finding informal caregivers willing to participate. While all of the patients restricted to NWB agreed to participate, not all of them had an informal caregiver available for an interview, or they did not want to burden their informal caregiver with an interview. As a result, we only had a small number of matched dyads. This may have led to differences in responses regarding encouragement from informal caregivers or preferences for group versus individual therapy between the caregivers and patients.

We mapped inductively derived themes onto the ICF framework after completing the initial analysis. Although themes were first identified through an inductive process, aligning them with predefined ICF domains may have influenced categorization and interpretation, potentially constraining the nuance of participants’ perspectives.

We conducted interviews at different locations, one of which was the workplace of the interviewer. Although we did not include patients for whom the interviewer was the primary physiotherapist, some participants recognized her as a therapist, which might have influenced their responses. The interviewer’s professional background as a physiotherapist may also have shaped the way certain questions were framed or interpreted. While this clinical expertise enriched the contextual understanding of responses, we acknowledge that it may have introduced subtle bias. To mitigate this, we employed a topic list that was reviewed by the research team and piloted prior to data collection.

## Conclusion

Physical activity during the NWB period should be individualized, as perceptions of what constitutes physical activity and how much therapy is needed vary. Most participants considered physiotherapy two to four times per week, with sessions of approximately 30 min, to be sufficient. These sessions should include strength training, and be complemented by patient-regulated exercise. The primary goals of engaging in physical activity are to regain walking ability and return home. We recommend that rehabilitation plans actively incorporate the perspectives of patients restricted to NWB and their informal caregivers, and that any discrepancies between these perspectives and those of healthcare providers are explicitly addressed.

## Data Availability

The datasets used and/or analyzed during the current study are available from the corresponding author on reasonable request.

## Appendix

See appendix Tables

**Table 2:** Semi-structured interview guide for non-weight-bearing patients

	Question
General	Could you briefly introduce yourself and tell me why you are currently staying at (location)?
	How physically active were you before you were restricted from bearing weight on your leg?
Knowledge	What do you know about being physically active during the non-weight-bearing period?
	What activities do you consider to be physical activity during this non-weight-bearing period?
Behavior	On average, how much do you move during a day in this non-weight-bearing period?
Motivation	What motivates you to be physically active?
	Are there physical activities for which you feel more or less motivated? What influences this?
Content (and environment)	What would be your preferred content and frequency of physical activity during this non-weight-bearing period?

2 and

**Table 3:** Semi-structured interview guide for informal caregivers

	Question
General	Could you briefly introduce yourself?
Knowledge	What do you know about being physically active during the non-weight-bearing period?
	What activities do you consider to be physical activity during this non-weight-bearing period?
Behavior	How do you support your relative in being physically active?
Motivation	What is the importance of being physically active during this period for your relative?
Content (and environment)	What do you consider to be the preferred content and frequency of physical activity during this non-weight-bearing period for your relative?

3
