# Correction: Exploring perspectives of older adults and informal caregivers on physical activity during non-weight-bearing rehabilitation: a qualitative study

**DOI:** 10.1007/s41999-026-01431-z

**Published:** 2026-02-18

**Authors:** Elma van Garderen, Mandy Visser, Wilco P. Achterberg

**Affiliations:** 1 Department of Public Health and Primary Care, Leiden University Medical Center, Hippocratespad 21, 2333 ZD Leiden, The Netherlands; 2 Topaz, Aaltje Noordewierlaan 50, 2324 KS Leiden, The Netherlands

**Correction: European Geriatric Medicine (2025) **10.1007/s41999-025-01390-x.

In the originally published version of this article, the Methods section incorrectly stated that “Eligible for inclusion were patients aged 65 + .”

During the study, the lower age limit for inclusion was revised to 60 years. Accordingly, one participant aged 61 years was included, as correctly reported in the Results section. However, after this decision was made, the change was not reflected in the final version of the Methods section.

The corrected sentence in the Methods section should read:

“Eligible for inclusion were patients aged 60+.”

This correction does not affect the results, interpretation, or conclusions of the study. The authors apologize for this oversight.

The Original Article has been corrected.

